# Differing needs of mothers and fathers during their child’s end-of-life care: secondary analysis of the “Paediatric end-of-life care needs” (PELICAN) study

**DOI:** 10.1186/s12904-020-00621-1

**Published:** 2020-08-04

**Authors:** Tanja Leemann, Eva Bergstraesser, Eva Cignacco, Karin Zimmermann

**Affiliations:** 1 School of Human Medicine, University of Zurich Faculty of Medicine, Zurich, Switzerland; 2 Paediatric Palliative Care and Children’s Research Center, University Children’s Hospital Zurich, Steinwiesstrasse 75, Zurich, 8032 Switzerland; 3 Department of Health Professions, Berne University of Applied Sciences, Berne, Switzerland; 4 Department Public Health – Nursing Science, University of Basel, Bernoullistrasse 28, Basel, 4056 Switzerland

**Keywords:** Paediatrics, Terminal care, End of life, Parents, Needs assessment, Surveys and questionnaires

## Abstract

**Background:**

Mothers and fathers are severely challenged when providing care for their terminally ill child at end of life. Caregiving needs have been studied predominantly in mothers. Differences in caregiving needs between mothers and fathers during their child’s end of life have not, however, been explored so far. This knowledge is of importance to best meet individual parental needs in paediatric end-of-life care.

**Methods:**

Secondary analysis of a quantitative survey on parental needs during their child’s last 4 weeks of life, collected in the Swiss multicentre “Paediatric End-of-Life Care Needs” (PELICAN) study. Caregiving needs of mothers and fathers (parental dyad) who had lost a child due to a cardiological, neurological or oncological disease or during the neonatal period in the years 2011–2012 were retrospectively assessed using a questionnaire representing six evidence-based quality domains of paediatric palliative and end-of-life care.

**Results:**

Seventy-eight parental dyads were included in this analysis. Differences between mothers and fathers were mostly found around needs to be supported as a family. In all, 28 out of 34 needs-related questionnaire items were scored higher by mothers than by fathers, indicating higher importance for that need to be met. The results indicate that these differences might relate to different caregiving roles and gender-specific coping strategies.

**Conclusions:**

To best meet parental needs in paediatric end-of-life care, particular attention should be paid to both mothers and fathers and their specific caregiving roles, as differences in these roles might influence their needs in this exceptional situation. Therefore, healthcare professionals should identify how parental dyads mutually navigate care for their sick child to best meet their needs in support. Additionally, mothers and fathers should be supported in their individual coping strategies.

## Background

An estimated 21 million children (0–19 years) worldwide are in need of palliative care, including more than 8 million children who require specialised paediatric palliative care (PPC) during their illness and at the end of life (EOL) [1]. PPC, a discipline still relatively new to modern healthcare, is a holistic approach to improving the quality of life of children with life-limiting and -threatening diseases and their families through active care of the body, mind and spirit as an entity [2]. Based on the values of family-centred care, PPC is driven by the needs of the patients as well as those of their parents and siblings [3]. To be able to provide individualised care, it is important for healthcare providers to be aware of the particular needs of parents during this vulnerable phase. Recent studies on parental perspectives in PPC or EOL care have shown that parental needs include sincere communication about the child’s condition, genuine relationships, continuity, coordination and accessibility of care, alleviation of suffering, emotional, spiritual and cultural support as well as bereavement support [4–6].

Caregiving needs might, however, differ between mothers and fathers. In the literature, traditional roles in the division of labour are described for parents caring for a child with a life-limiting disease. Mothers are thus referred to as primary caregivers, staying with their child during hospitalisation, while fathers are referred to as breadwinners assuring the financial income [7–9]. In consequence, mothers were regarded as having higher needs for dealing with the medical aspects of their child’s illness than were fathers [7]. Due to a gender imbalance occurring in research, findings about parental experiences and needs in PPC predominately represent the mothers’ perspective [10]. A study focusing on the fathers’ experiences of children with a life-limiting illness showed that the paternal role was more practical than emotional, leading to a more pragmatic coping strategy [11]. Furthermore, fathers’ conflicts between work requirements and their desire to be with the child were reported [11]. Although this literature reflects a heteronormative traditional family set up, which is certainly not representative for the entire population internationally, it remains the predominant family structure in many countries such as Switzerland, where this study was conducted [12]. Regarding the grieving process among parents of deceased children, gender-specific differences have been identified as well. Mothers preferred social support and opportunities to exchange their experiences, whereas many fathers reported that distracting themselves from their thoughts and feelings by returning to work was more helpful [13, 14]. Recently, conditions and events occurring during the EOL phase of a child with cancer were found to contribute to prolonged grief, and were experienced differently by fathers than by mothers [15].

Based on this existing evidence related to parenting roles, coping and bereavement, relevant differences in caregiving needs between mothers and fathers might be present during their child’s EOL phase. To our knowledge, no study has yet described gender specific differences in caregiving needs during that time. The aim of this study was, therefore, to explore differences in caregiving needs during a child’s EOL phase between mothers and fathers.

## Methods

### Study context and design

This study used secondary data from the nationwide multicentre “Paediatric End-of-Life Care Needs” (PELICAN) study in Switzerland [16]. The PELICAN study reported comprehensive information about paediatric EOL care practices (PELICAN I) [17] as well as the perspectives of families (PELICAN II) [18] and health professionals (PELICAN III) [19]. For the present study we analysed questionnaire data from the PELICAN II study, which retrospectively assessed bereaved parental experiences and needs during a child’s EOL care (defined as the last 4 weeks of life for the PELICAN study) [18]. Access to the raw data from the PELICAN study was granted by the study’s principal investigators and co-authors of this paper (E.B. and E.C.).

### Participants and recruitment

Parents (mothers and fathers) of children (0–18 years) who had died in Switzerland in the years 2011 or 2012 due to a cardiological, neurological, or oncological disease or during the neonatal period were identified, using administrative hospital death data. Parents were excluded if their child had died during the first 24 h of life or if parents were not able to read and understand German, French or Italian. Recruitment took place in 17 hospitals, 2 long-term institutions, and 4 community care centres between July 2013 and March 2014. The PELICAN study received approval from Human Research Committees from the 11 Swiss cantons where the recruiting institutions were situated (leading committee: ethics committee of canton Zurich, KEK ZH Nr. 2012–0537). An invitation letter together with informed consent documents were sent to 267 families. Of these, 135 families (51%) consented to receive the study questionnaire. Further details of the recruitment processes have been described elsewhere [18].

### Data collection

The Parental PELICAN Questionnaire (PaPEQu) was sent out in April 2014 to mothers and fathers who consented to participate in the PELICAN II study. This study questionnaire was developed to assess bereaved parental experiences and needs related to their deceased child’s EOL care. It is structured according to six evidence-based quality domains of family-centred EOL care: *support of the family unit*, *relief of pain and other symptoms*, *continuity and coordination of care, communication*, *shared decision making* and *bereavement support.* Within each of these six domains, scale items targeting parental experiences and scale items targeting parental needs were used. The PaPEQu exists in four slightly different versions, accounting for differences between characteristics of the four diagnostic study patient groups. Because of this, the item count ranged from 91 to 95, with the addition of 13 socio-demographic items. Initial validity and reliability of the PaPEQu has been demonstrated and reported along with a detailed description of its development [20]. In this study only the 34 needs-related items representing the six evidence-based quality domains were used and analysed for gender-specific differences, as well as 7 socio-demographic items. A 7-point scale (1 to 7) with endpoint anchors ‘not important at all - very important’ was used. For four items the response option ‘not applicable’ was available. See Table 2 for a complete list of the needs-related items.

### Data analysis

All study items were analysed by applying descriptive and explorative statistics using measures of central tendency and dispersion, and frequencies with percentages. Only cases with responses from both mothers and fathers of the same child were included. Differences within the parental dyads were analysed using the Wilcoxon signed-rank test. A Bonferroni corrected probability of ≤0.001 was set to decide statistical significance. IBM® SPSS® Statistics 24 for Microsoft® was used to perform the analyses.

## Results

### Sample characteristics

Of the 200 mothers and fathers who completed the PaPEQu, 156 questionnaires could be paired into 78 parental dyads and were included for analysis. All but one mother and one father (*n* = 154, 99%) were married or in a partnership at the time of the survey. Table 1 shows a complete description of the sample. Thirty-one mothers (40%) and 30 fathers (39%) reported that their own health was negatively influenced by the illness and death of their child. Differences between mothers and fathers were observed regarding their employment status at the time of the child’s death, while the education level of mothers and fathers were similar. Neonates comprised the largest represented diagnostic group (*n* = 68, 44%), followed by children suffering from neurological (*n* = 36, 23%), oncological (*n* = 34, 22%) and cardiological (*n* = 18, 11%) conditions, respectively. Seventeen (22%) of the neonates/children and their families received specialised palliative care and 40 (51%) spent at least 1 day at home during their last four weeks of life. Of these 40, 19 (48%) were supported by community care services during their days at home.

**Table 1:** Sample characteristics of parental dyads *(N =* 156*)*

Characteristics	Total *N =* 156 (100%)	Mothers *n =* 78 (50%)	Fathers *n =* 78 (50%)
Age^a^, *M (SD)*	40 (6.7)	39 (6.1)	42 (7.1)
Language, *n (%)*			
German	131 (84.0)	66 (84.6)	65 (83.3)
French	18 (11.5)	9 (11.5)	9 (11.5)
Italian	7 (4.5)	3 (3.8)	4 (5.1)
Education, *n (%)*			
School levels^b^	6 (3.8)	1 (1.3)	5 (6.4)
Post school education^c^	70 (44.9)	39 (50.0)	31 (39.7)
Tertiary level^d^	56 (35.9)	28 (35.9)	28 (35.9)
University degree	24 (15.4)	10 (12.8)	14 (17.9)
Employment status at death of the child, *n (%)*			
Working	76 (48.7)	24 (30.8)	52 (66.7)
Off work^e^	80 (51.3)	54 (69.2)	26 (33.3)
Family income^f^, *n (%)*	*N* = 130		
≤ CHF 100,000.-	62 (47.7)		
> CHF 101,000.-	68 (52.3)		

^a^Age at the time of the survey. ^b^Consists of primary and secondary level. ^c^Consists of college and vocational education. ^d^Consists of degrees from schools of higher education. ^e^Consists of being on sick leave, on unpaid leave, being unemployed or in educational training. ^f^Annual gross pay, Swiss average lies at CHF 152,000 [21]

Except for the item *family income*, which showed a missing value of 17% due to the response option of not answering the question, no further information was missing for the socio-demographic items. For the needs-related items, missing values ranged between 0 and 51%. However, for four items the response option ‘not applicable’ was given and chosen. Not counting those answers, true missing values ranged between 0 and 23%.

### Parental needs

**Fig. 1:**
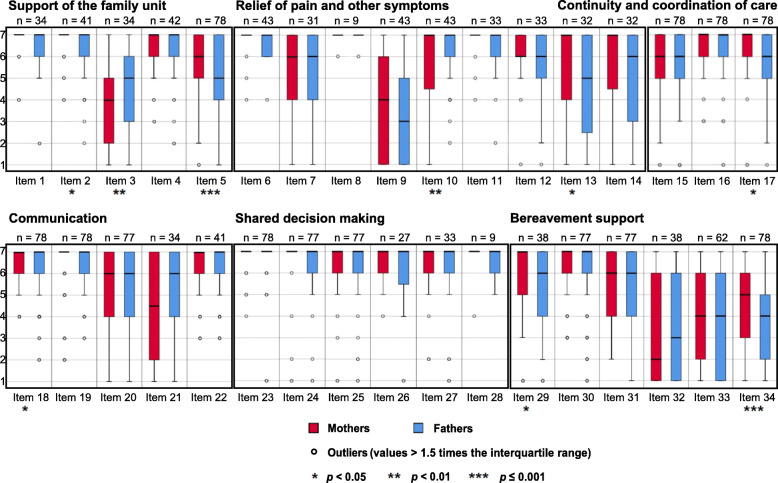
Distribution of mothers' and fathers' responses across all six evidence-based quality domains

In all, 28 out of 34 needs-related items (82%) were scored higher by mothers than fathers. Statistically significant differences between mothers and fathers were found in 2 items (6%, *p* ≤ 0.001); see Fig. 1 for an overview of distribution and differences, and Table 2 for a complete list of the needs-related items.

**Table 2:** Complete list of needs-related items

Domains and items	Presence in questionnaire version			
	Cardiology	Neonatology	Neurology	Oncology
Support of the family unit domain: I needed				
1. To be involved in my child‘s care			x	x
2. To have a place to sleep in the hospital close to my child^a^	x	x		
3. To have respite from the care of my child			x	x
4. To have a room where my family and I could spend some private time together	x	x		
5. To share my fears and worries with someone from the healthcare team	x	x	x	x
Relief of pain and other symptoms domain: I needed				
6. That my child received enough medication to ease her/his suffering	x		x	x
7. That my child was awake and receptive enough to be able to play/speak/or do things with us or other people around			x	x
8. To take my child in my arms	x			
9. That my child received complementary and alternative medicine	x		x	x
10. That my child received fluids until the end	x		x	x
11. To have physical contact with my child		x		
12. That my child received medication to calm her/him		x		
13. To be able to use non-pharmacological measures to ease my child’s suffering e.g. massage, tucking		x		
14. That I could give my child milk, either through the tube, with a bottle or a cotton swab		x		
Continuity and coordination of care domain: I needed				
15. To have a professional from the healthcare team to coordinate the care of my child	x	x	x	x
16. To have the same physician providing care	x	x	x	x
17. That my child’s care was mostly provided by the same nurses	x	x	x	x
Communication domain: I needed				
18. To have the opportunity to ask questions at all times	x	x	x	x
19. To be continuously informed about my child’s condition	x	x	x	x
20. To find out how my child would die	x	x	x	x
21. To be supported in maintaining hope despite the hopeless situation			x	x
22. To be informed early about my child’s imminent death	x	x		
Shared decision making domain: I needed				
23. To be involved in taking decisions	x	x	x	x
24. That my personal beliefs and values were considered when taking decisions	x	x	x	x
25. Not to have the feeling that I had to take decisions all by myself	x	x	x	x
26. That the cessation of non-helpful treatments was discussed with me			x	x
27. That the cessation of life-sustaining measures was discussed with me		x		
28. That the measures to resuscitate my child were discussed with me	x			
Bereavement support domain: I needed				
29. To have the choice of where child might die^a^	x	x	x	x
30. That family and friends could say goodbye to my child	x	x	x	x
31. That I was supported by the healthcare team to structure the hours after the death of my child according my needs	x	x	x	x
32. To take my child home after her/his death so that family and friends could say goodbye^a^	x	x	x	x
33. That someone from the healthcare team attended my child’s funeral or burial^a^	x	x	x	x
34. To stay in contact with someone from the healthcare team after my child’s death	x	x	x	x

^a^ Response option ‘not applicable’ available

#### Support of the family unit

Only the need *‘to have respite from the care of my child’* (*Mdn* = 5.0, *range* = 1–7 vs. *Mdn* = 4.0, *range* = 1–7) was scored higher by fathers than mothers. Both mothers and fathers rated the item *‘to be involved in my child’s care*’ (*Mdn* = 7.0, *range* = 2–7) highest and the item *‘to have respite from the care of my child’* (*Mdn* = 4.0, *range* = 1–7) lowest.

#### Relief of pain and other symptoms

The two needs *‘that my child was awake and receptive enough to be able to play/speak/or do things with us or other people around’* (*Mdn* = 6.0, *range* = 1–7 vs. *Mdn* = 6.0, *range* = 1–7) and *‘that my child received fluids until the end’* (*Mdn* = 7.0, *range* = 2–7 vs. *Mdn* = 7.0, *range* = 1–7) were scored higher by fathers than mothers. Both mothers and fathers rated the item *‘to take my child in my arms’* (*Mdn* = 7.0, *range* = 6–7) highest and the item *‘that my child received complementary and alternative medicine’* (*Mdn* = 3.0, *range* = 1–7) lowest.

#### Continuity and coordination of care

All items were rated higher by mothers. Both mothers and fathers scored the need *‘to have the same physician providing care’* (*Mdn* = 7.0, *range* = 1–7) highest and the need *‘to have a professional from the healthcare team to coordinate the care of my child’* (*Mdn* = 6.0, *range* = 1–7) lowest.

#### Communication

The need *‘to be supported in maintaining hope despite the hopeless situation’* (*Mdn* = 6.0, *range* = 1–7 vs. *Mdn* = 4.5, *range* = 1–7) was scored higher by fathers than mothers. Mothers showed highest scores for the item *‘to have the opportunity to ask questions at all times’* (*Mdn* = 7.0, *range* = 4–7) and lowest scores for the item *‘to be supported in maintaining hope despite the hopeless situation’* (*Mdn* = 4.5, *range* = 1–7). Fathers rated the need *‘to be continuously informed about my child’s condition’* (*Mdn* = 7.0, *range* = 3–7) highest and the need *‘to find out how my child would die’* (*Mdn* = 6.0, *range* = 1–7) lowest.

#### Shared decision making

The need *‘that the measures to resuscitate my child were discussed with me’* (*Mdn* = 7.0, *range* = 5–7 vs. *Mdn* = 7.0, *range* = 4–7) was scored higher by fathers than mothers. Both mothers and fathers showed highest scores for the item *‘to be involved in taking decisions’* (*Mdn* = 7.0, *range* = 1–7). Mothers rated the need *‘not to have the feeling that I had to take decisions all by myself’* (*Mdn* = 7.0, *range* = 1–7) lowest, while fathers rated the need *‘that the cessation of non-helpful treatments was discussed with me’* (*Mdn* = 7.0, *range* = 1–7) lowest.

#### Bereavement support

Only the need *‘to take my child home after her/his death so that family and friends could say goodbye’* (*Mdn* = 3.0, *range* = 1–7 vs. *Mdn* = 2.0, *range* = 1–7) was scored higher by fathers than mothers. Both mothers and fathers showed highest scores for the item *‘that family and friends could say goodbye to my child’* (*Mdn* = 7.0, *range* = 1–7). Mothers rated the need *‘to take my child home after her/his death so that family and friends could say goodbye’ (Mdn* = 2.0, *range* = 1–7) lowest, while fathers rated the need *‘to stay in contact with someone from the healthcare team after my child’s death’* (*Mdn* = 4.0, *range* = 1–7) lowest.

## Discussion

To our knowledge, this is the first study to explore differences in the caregiving needs of bereaved mothers and fathers regarding their child’s EOL care. The study indicates that these differences might relate to different caregiving roles and coping strategies. To mothers it is more important to be able to stay close to their child at night, to be able to ask questions all the time and to have the same nurses providing care. Fathers, facing conflicts between work and care requirements, have a greater need for respite from care. Our findings show that the majority of the items were scored higher by mothers, probably indicating that the majority of the stated needs were more important to mothers than to fathers. Substantial differences were mostly identified in the questionnaire domain *support of the family unit,* i.e. needs for support as a family.

### Support of the family unit

Differences in this domain might be related to the different caregiving roles of mothers and fathers, leading to divergent needs for support. Even though the specific caregiving roles of mothers and fathers were not explored in this study, it can be assumed that traditional family structures with mothers as primary caregivers and fathers as breadwinners are predominant in Switzerland and in this study’s sample. Federal statistics on time spent on paid work and family workload for mothers and fathers in Switzerland generally reflect the gendered roles of mothers as regular caretakers of children and fathers as breadwinners [22]. The parents’ employment status at the time of the child’s death supports our assumption. Even though this family structure might not be the norm internationally, it is most commonly described in the literature concerned with parental needs of severely ill children [7–9]. Also, the age distribution and marital status represented in our study sample are in line with other research in the field [23].

As to needs, mothers whose main focus is caregiving want to spend every minute possible with their dying child [24]. Therefore, their need for a bed next to their child’s might be greater. Since mothers spend more time in the hospital, their relationship with the healthcare team is often closer than that of fathers. Due to their constant presence with their child, mothers might need a close confidant from the healthcare team with whom they can talk about their child’s medical conditions as well as their emotions. For this reason, the need for sharing fears and worries with a member of the healthcare team might be more important to mothers than to fathers. It is not surprising, then, that it was also more important for mothers than for fathers that their child’s care was mostly provided by the same nurse. Even though mothers are more involved in the child’s care, fathers expressed a greater need for respite from care. This might be due to excessive demands involving conflicts between work requirements and care of the sick child [11]. Furthermore, fathers might not wish respite for themselves but rather for their partner. It was reported that fathers often tried to encourage their partners to take time for themselves [8]. Fathers also reported concerns about decreased partner intimacy due to lack of time to maintain the partnership during care for a sick child [11]. This might be another explanation for why fathers in our study rated the need to have respite from the care of their child as significantly more important than did mothers.

### Coping strategies of mothers and fathers

Coping with one’s child’s EOL places a heavy burden of distress on parents. According to the theory of stress and coping of Lazarus and Folkman [25], modified by Folkman several years later [26], the stress of caregiving requires coping to regulate distress. Although coping as a response to distress involves negative emotions, it can also, in parallel, regularly evoke positive emotions, which are sought and created in order to provide momentary relief from distress, but do not indicate the conclusion of coping activity. Folkman describes three pathways that can lead to these positive psychological states: a) positive reappraisal and meaning-making achieved by interpreting the situation in terms of deeply held values and beliefs; b) revising goals and planning goal-directed problem-focused coping, and c) activating spiritual beliefs and experiences to find existential meaning [26]. It is well known that mothers and fathers cope differently in response to stressful events, indicating that mothers often prefer social support and opportunities to exchange their experiences and emotions [13, 14]. It is reported that fathers are more likely to take care of themselves than are mothers, who tend to neglect their own needs and comfort [8]. For fathers, it was more important to be supported in keeping up hope than it was for mothers. Maintaining a positive attitude is referred to as a pragmatic coping strategy preferred by fathers. Moreover, it is possible that fathers do not want their partners to be confronted with hopelessness because they want to protect them [11].

Concerning the relief of pain and other symptoms, being able to use non-pharmacological measures and complementary medicine was more important to mothers than fathers on the one hand. A possible reason for this might be that women have a more positive attitude to alternative medicine and are more likely to use it as a therapy for themselves than are men [27]. On the other hand, it was more important for fathers that the child should receive fluids until the end. The paternal coping strategy of preferring to act instead of waiting and watching might be a possible explanation here [11].

It was more important for mothers than for fathers to have a choice concerning the child’s place of death. Mothers who had lost their child described finding some peace in being able to arrange their child’s death [28]. Having good memories of their child’s death was important, and positively influenced their grief process [24]. As also reported elsewhere, mothers and fathers felt that they had lost their second home after their child’s death, as contact with the healthcare team ended abruptly [29]. As mothers usually spend more time in hospital, their feeling of abandonment after the child’s death might be greater than that of fathers. It has been shown that mothers tend to bond more strongly with their child than do fathers, which could create tension between different coping strategies within a couple [13]. For this reason, mothers might continuously seek opportunities to stay in contact with members of the healthcare team who knew their child, to talk about their burden and to share their memories with them [13, 14, 24]. This was a difference between mothers and fathers that was also significantly reflected in our study.

### Strengths and limitations

This study provides quantitative evidence from 156 bereaved mothers and fathers of differing needs during their child’s EOL care, covering six evidence-based quality domains for family-centred care. Previous research on caregiving differences between mothers and fathers in PPC is scarce. So far, the mothers’ perspectives have been overrepresented in research due to gender imbalances. This study adds knowledge to that domain by presenting the perspectives of 78 fathers and 78 mothers (parental dyads).

Nevertheless, there are several limitations. The ratings of most items were high, with a skewed sample distribution showing ceiling effects. This led to limited variability and few significant differences within the parental dyads. Additionally, the retrospective nature of this cross-sectional study might have introduced a recall bias. The study focused on parental caregiving needs during a child’s last 4 weeks of life; needs, therefore, might have been different in the child’s earlier illness trajectory. It has been reported before that parental concordance increases during the time of their child’s illness [30]. Further, the recruitment and informed consent approach might have induced a selection bias, as only parents who consented to participate received the study questionnaire. This might have resulted in the inclusion of participants with rather favourable experiences, also affecting their reported needs. Further, it might have led to a study sample of mothers and fathers living in a stable partnership at the time of completing the questionnaire – a potential bias, but also the norm in Switzerland. Finally, the representativity of the sample is limited by the fact that Switzerland’s non-French, −German, or -Italian speaking migrant population was excluded. Therefore, generalisability to other countries is limited as well.

## Conclusions

To best meet parental needs in paediatric EOL care, particular attention should be paid to both mothers and fathers and their specific caregiving roles, as differences in caregiving roles might influence their needs. Therefore, healthcare professionals should identify how parental dyads mutually navigate care for their sick child in order to appropriately meet their needs in support. Additionally, mothers and fathers should be supported in their individual coping strategies. Mothers, when primary caregivers, often have higher needs for a permanent exchange with members of the healthcare team, to talk about their child’s medical condition as well as their own emotions. Fathers often favour more pragmatic options of coping, such as retaining hope. These gender-specific strategies should be acknowledged when providing care around a child’s EOL and beyond.

As this survey has been the first to quantitatively identify differences in caregiving needs between mothers and fathers regarding their child’s EOL care, our findings and their interpretations should be further explored in other samples and with mixed methodological approaches. Especially the perspectives and needs of parents living in non-traditional family structures should be  emphasised. Only the appreciation and understanding of existing family structures and caregiving roles, as well as gender-specific differences will lead to improvements in the support of mothers and fathers during this vulnerable period.

## Data Availability

The datasets analysed during this study are not publicly available due to ongoing analyses but are available from the corresponding author on reasonable request.

## Abbreviations

EOL: End-of-life
PaPEQU: Parental PELICAN questionnaire
PELICAN: Paediatric end-of-life care needs
PPC: Paediatric palliative care
